# The suboccipital, telovelar, transsuperior fovea approach to dorsal pontine lesions

**DOI:** 10.3171/2019.7.FocusVid.1927

**Published:** 2019-07-01

**Authors:** M. Yashar S. Kalani, Kaan Yağmurlu, Nikolay L. Martirosyan, Robert F. Spetzler

**Affiliations:** Department of Neurosurgery, Barrow Neurological Institute, St. Joseph’s Hospital and Medical Center, Phoenix, Arizona

**Keywords:** brainstem, cavernous malformation, pontine, safe entry zone, surgery, video

## Abstract

Dorsal pons lesions at the facial colliculus level can be accessed with a suboccipital telovelar (SOTV) approach using the superior fovea safe entry zone. Opening the telovelar junction allows visualization of the dorsal pons and lateral entry at the level of the fourth ventricle floor. Typically, a lateral entry into the floor of the fourth ventricle is better tolerated than a midline opening. This video demonstrates the use of the SOTV approach to remove a cavernous malformation at the level of the facial colliculus. This case is particularly interesting because of a large venous anomaly and several telangiectasias in the pons. Dissections in the video are reproduced with permission from the Rhoton Collection (http://rhoton.ineurodb.org).

The video can be found here: https://youtu.be/LqzCfN2J3lY.

## Transcript

This case is a suboccipital telovelar approach used for resection of a dorsal pontine cavernous malformation. This 24-year-old female presented with left facial droop, facial numbness, gait difficulties, and a history of several bleeds caused by this pontine cavernous malformation located in the dorsal brainstem. The magnetic resonance imaging demonstrates the classic appearance of a cavernous malformation located eccentric to the left side in the dorsal pons with significant telangiectasias present throughout the pons as well as a large developmental venous anomaly. We elected to approach this lesion using a midline suboccipital craniotomy and a telovelar extension to resect the cavernous malformation.

### 1:05 Anatomical dissection of the exposure

After performing a suboccipital craniotomy, the telovelar junction is exposed by dissecting the arachnoid adhesions connecting the cerebellar hemispheres and the cerebellar vermis. These anatomical dissections expose the telovelar junction. For resection of dorsal pontine lesions at the level of the facial colliculus, a lateral entry into the brainstem using the superior fovea has been described as a safe entry zone. That is what was used to approach the lesion in this case. Another anatomical dissection demonstrates the operative view obtained after a wide suboccipital craniotomy. For lesions that are high-riding in the dorsal pons, a significant opening in the caudal direction is necessary for the surgeon to obtain the necessary view rostrally to approach these lesions. This anatomic dissection demonstrates the local anatomy at the level of the facial colliculus. Approaches at the midline are less well tolerated in the dorsal pons than those approaches that are placed slightly off midline. Again, for this lesion and for other lesions located at the level of the facial colliculus, using the superior fovea has been described as a safe entry zone.

### 2:16 Patient positioning

The patient is placed prone, and the intraoperative photo demonstrates the skin incision necessary for performing this suboccipital approach. The dotted line demonstrates the possible extension necessary in the caudal direction so that the surgeon may obtain the rostral view necessary to approach this lesion. A significant tucking of the chin and flexion of the neck is necessary for these cases. After performing a midline suboccipital craniotomy, the cerebellar hemispheres are visualized. The arachnoid overlying the cisterns are opened, and cerebrospinal fluid release is performed to obtain cerebellar relaxation.

### 2:54 Exposing the telovelar junction

In this case, it is important to open the telovelar junction, exposing the foramen of Luschka. This is critical for obtaining the off-lateral trajectory necessary to enter this cavernous malformation. Once the telovelar craniotomy is performed, the neuronavigation is utilized to obtain a point of ideal entry into the brainstem to resect the cavernous malformation. At the doral pons, several safe entry zones have been described to resect lesions deep to the pial surface. We elected to utilize a superior fovea safe entry zone, given that this lesion was located at about the level of the facial colliculus. A small opening is placed into the brainstem and, by stretching the opening, is enlarged just enough for a suction and a microinstrument to be placed within the opening to resect the cavernous malformation. The classic appearance of the cavernous malformation is apparent. The hemosiderin staining demonstrates that this lesion has bled in the past.

### 4:05 Cavernous malformation removal

The cavernous malformation caverns are resected in a piecemeal fashion using toothed microforceps. The toothed microforceps are critical because they allow the surgeon to peel the cavernous malformation from adjacent eloquent tissue, therefore minimizing injury to this tissue. This technique also minimizes the use of cautery. At the completion of the procedure, hemostasis is performed. The patient’s exam was neurologically stable without a significant change in the patient’s facial function. Postoperative magnetic resonance imaging demonstrates complete removal of the cavernous malformation with preservation of the developmental venous anomaly. This last magnetic resonance imaging, which is a FLAIR sequence, also nicely demonstrates the path of entry into the cavernous malformation. This path of entry is eccentric to the left side and off midline from floor of the fourth ventricle. The telovelar craniotomy is important and necessary to be able to obtain this lateral view necessary to obtain the resection of the lesion.^1–5^
